# Atomic Botany: The Botanical Career of Janice Carson Beatley, and the Flora, Vegetation, and Ecology of the Nevada Test Site in Cold War United States (1959–1973)

**DOI:** 10.1007/s10739-025-09819-6

**Published:** 2025-05-20

**Authors:** Tod F. Stuessy, Ronald Pilatowski

**Affiliations:** The Ohio State University, Columbus, Ohio USA

**Keywords:** Janice Carson Beatley, Atomic Energy Commission, Nevada Test Site, Women botanists, Plant ecology

## Abstract

This paper focuses on the career of Janice Carson Beatley (1918–1987), a botanist and plant ecologist, who moved into the male-dominated field of nuclear studies to investigate the flora, vegetation and ecology of the Nevada Test Site between 1959 and 1973. It examines her background, training, her employment history, and her relationship with plant ecologist John “Jack” Wolfe, who himself had accepted a position in the Atomic Energy Commission (AEC) in 1958. It explores her scientific work, which included creating an inventory of the flora and vegetation of the region, but also assessing the damage from radioactive fallout, and inferring basic ecosystem processes in this desert region, which covered parts of the Mojave and Great Basin Deserts. She made major contributions to understanding these ecosystems, especially the precise role of precipitation as an environmental trigger for the development of the vegetation. By the end, she had established some sixty-eight permanent plots and developed a herbarium of approximately 13,500 mounted specimens, many of which were deposited at the National Museum of Natural History at the Smithsonian Institution in Washington, DC. Toward the end of her work with the AEC, she became embittered by a sense of having been marginalized and not accorded proper appreciation for her scientific contributions. Her career illustrates difficulties that confronted women scientists as they attempted to establish themselves in fields dominated by men, such as nuclear energy.

## Introduction

The brief period of euphoria in 1945 after the close of the Second World War in Europe was soon replaced by intense competition with the Soviet Union and the beginning of the Cold War. Of particular concern was the development of atomic weapons, highlighted as militarily significant after the US dropped atomic bombs on Hiroshima and Nagasaki, which ended the war in the Pacific. The USSR was rapidly developing its own atomic bombs,^1^ encouraging politicians and military leaders in the US to push aggressively forward developing even more powerful weapons.

The conception and manufacture of new atomic bombs required periodic testing as a means of confirming theoretical efficacy. The original atmospheric test was the Trinity plutonium detonation, which took place on July 16, 1945 in New Mexico on the Alamogordo Bombing Range. Due to concerns of potential harm to neighboring populations from radioactive fallout, new nuclear tests shifted in 1946 to the isolated Marshall Islands in the Pacific Proving Grounds (e.g., Bikini Atoll). Explosions in these islands created health hazards for Indigenous peoples in the region (Simon et al. 2006), and were also logistically complex for American personnel and equipment. This led to a search for a new continental US site to conduct further testing. After considerable discussion, consideration of alternative sites, and political wrangling, the decision was made for further atomic tests to be carried out in southeastern Nevada, in the Nevada Proving Grounds of the US Army. This area was designated in December of 1950 as the Nevada Test Site (NTS) and publicly announced by President Truman on January 12, 1951 (Fehner and Gosling 2000).

**Fig. 1:**
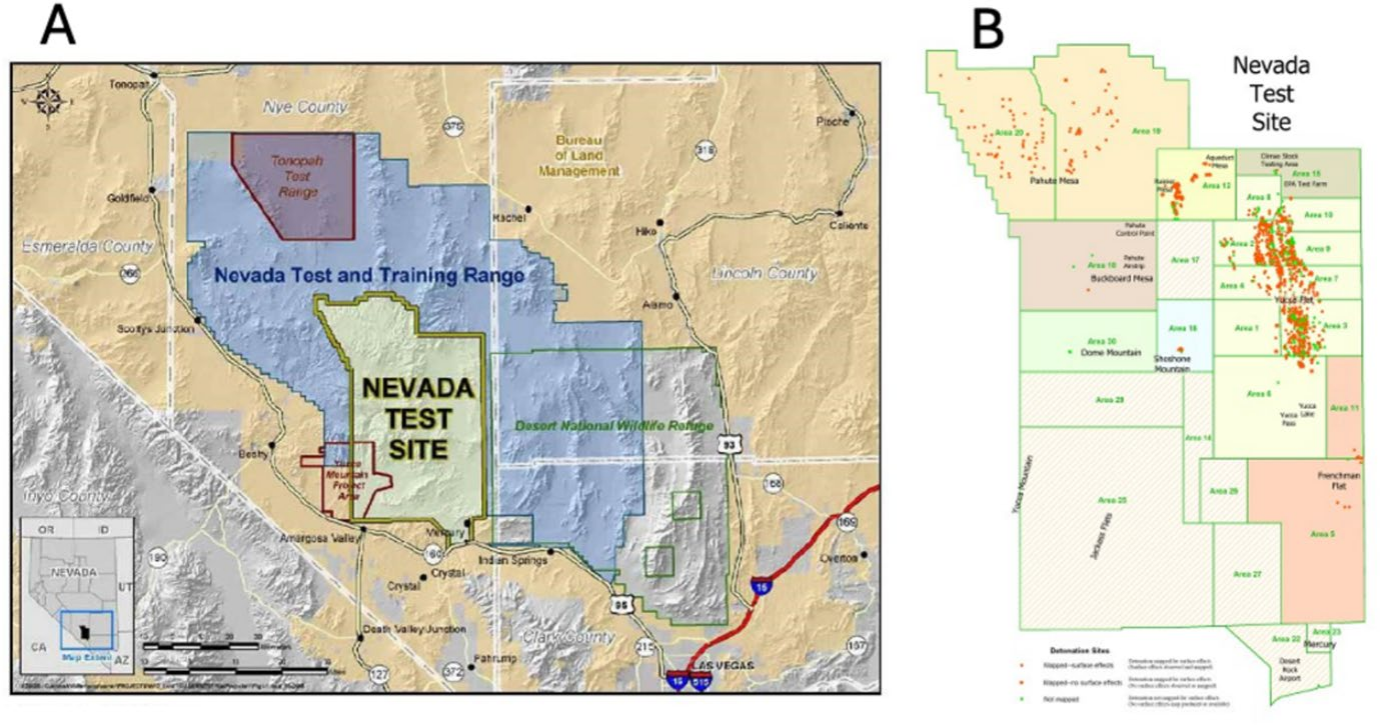
The Nevada Test Site in southeastern Nevada, showing general location (A), and (B) designated areas, major geographic features, and locations of nuclear detonations. A from National Cancer Benefits Center website; B, from Wikipedia

The NTS (Fig. 1; now the Nevada National Security Site) became the permanent site for thermonuclear testing for the US and from 1951–1962 more than 100 atmospheric tests were conducted there (Gowin 2019, p. 149). After the Comprehensive Nuclear Test Ban Treaty banned atmospheric testing in 1962, approximately 900 additional underground tests were carried out up to September 23, 1992. Some subcritical, that is, non-explosive tests were conducted after this time. During these numerous tests, concern began to grow regarding potential health hazards from radioactivity to animals, plants, and also to people, especially considering that Las Vegas was only 65 miles southeast.^2^ As public concerns regarding health implications from test site activities began to grow, the Atomic Energy Commission (AEC), which had been given civil jurisdiction over the atomic program after the Second World War, initiated many studies, including the effects of thermonuclear blasts and radioactive damage on buildings and other structures as well as on plants and vegetation at the NTS. The earliest studies were carried out by scientists in the Department of Biology at Highlands University in Las Vegas, New Mexico, located north of the original Trinity blast site.^3^ With development of the NTS and numerous additional atomic atmospheric detonations, these investigators briefly turned to analyze disturbances to the biota.^4^ In 1960, responsibility for research at the NTS shifted to the Laboratory of Nuclear Medicine and Radiation Biology of the University of California, Los Angeles (UCLA).

**Fig. 2:**
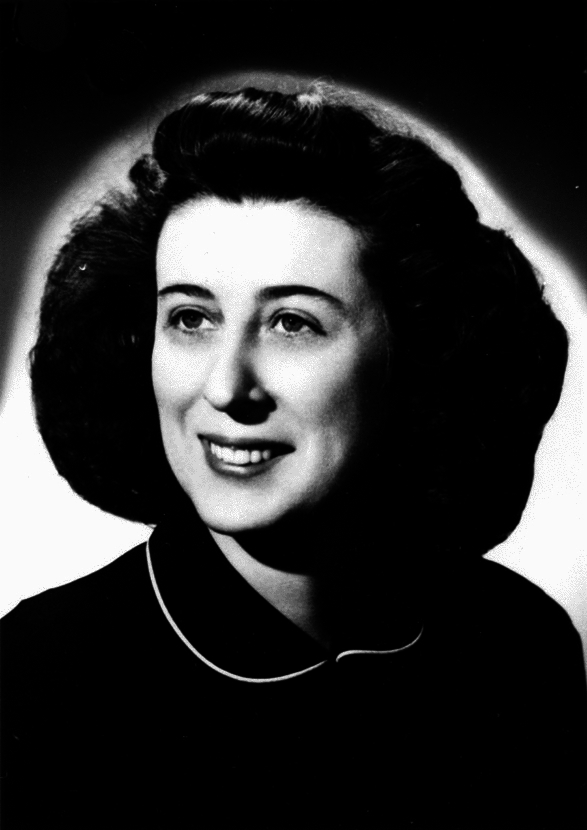
Janice Carson Beatley (1918–1987), about 1940. Stuckey Herbarium Archives

It was into these two academic departments at Highlands University and UCLA, both dealing with ecological impacts from the US atomic testing program, that Janice Carson Beatley (Fig. 2) began a professional association that would last for the major part of her scientific career. Although she began her career by studying the plants, vegetation, and ecology of the eastern deciduous forest, Beatley is perhaps best-known today for her contributions to these same aspects in southeastern Nevada in the Mojave and Great Basin Deserts. She undertook to inventory the vascular plants and vegetation of the NTS and to reveal the ecological dynamics in these desert regions, which involved establishing sixty-eight permanent plots that are still being monitored today (Webb et al., 2003). Other workers, mostly men (the only exception being Lora Shields), carried out ecological research at the NTS,^5^ but Janice Beatley was especially insightful and persevering.

Beatley’s career from 1953–1983 illustrated the challenges facing women scientists who had to compete with men for stable academic positions. Despite the fact that university-based research programs in the US were becoming more receptive to women’s scientific contributions, enabling some to distinguish themselves, few were able to obtain permanent academic positions. As Margaret Rossiter has shown, anti-nepotism policies laid down additional barriers (Rossiter 1982, pp. 195–197). Women at that time were still expected to marry and have children, leaving professional occupations to husbands and single men. Despite the high level of participation by women in the workforce during the war, men returning home sought their old or new jobs, and women were encouraged to return to their homes, which many of them did (Eisenmann 2006).

Janice Beatley was no different. After receiving her PhD from Ohio State University in 1953, she held many temporary academic and contractual positions, and despite strong academic achievements, she was unable to land a permanent academic post until the very end of her career. 6 This article focuses on her involvement and contributions to nuclear energy studies in the US, a field that was male-dominated. It explores her scientific contributions but also highlights the challenges encountered by women in achieving status equal to their male peers.

## Early Professional Development

Janice Carson Beatley was born on March 18, 1918, in Columbus, Ohio, the oldest child of Earle and Alice Elizabeth (Carson) Beatley (Stuckey 1990). She attended primary and secondary school in Columbus, graduating from North High School in 1935. She then furthered her education at The Ohio State University, obtaining a BA in 1940 (*cum laude*), majoring in zoology, after which she taught courses in algebra, biology, chemistry, general science, and physics at McArthur High School in McArthur, Ohio from 1943–1945.

Upon completion of her second year of teaching in 1945, Beatley enrolled as a graduate student in botany at The Ohio State University (OSU) to work with John (“Jack”) N. Wolfe (1910–1974), a plant ecologist. Wolfe served as advisor for her master’s thesis on the wintergreen herbaceous angiosperms of Ohio, and for her doctoral work on the primary forests of Vinton and Jackson Counties, Ohio.^7^ These studies continued the tradition of vegetational studies in Ohio established by Homer Sampson and Edgar Transeau at OSU (Stuckey 1992a, b). While a graduate student, Beatley was employed as an assistant instructor, both half-time and full-time, for a total of twenty-eight sections of General Botany between 1946 and 1953.

After receiving her PhD, Beatley took temporary positions over the next several years at a number of institutions. These included: instructor in the Botany Department at the University of Tennessee, Knoxville (1952–1953); instructor in Botany and Plant Pathology at OSU (1953–1954); instructor at the University of Tennessee in Botany for the summers of 1954 and 1955, while also taking on the rank of assistant professor in the Science Department at East Carolina College in Greenville, North Carolina; instructor again at OSU (1955–1956); acting assistant professor in ecology (1956–1957) at North Carolina State University; and acting assistant professor at the University of Tennessee (1959–1960). It is not surprising that Beatley had few publications during this period of her professional life, as she was moving around constantly. These jobs also were primarily teaching positions, giving her little time for research. In a narrative of her employment history she explicitly noted the influence of her gender: “continuing appointments were openly denied me because I was a woman.”^8^ But she also took appointments only in “first-quality institutions” to stay alive professionally. It is worth noting that when Beatley was seeking full-time academic and research appointments in the early 1950s, she encountered a wave of men earning advanced degrees in science after the Second World War with support from the GI Bill. While the number of women earning advanced degrees in science remained relatively stable over these years, the number of men taking the same degrees rose dramatically (Rossiter 1995, pp. 27–49). Competition for the academic openings of the type sought by Beatley was therefore strong.

## The Influence of Jack Wolfe

**Fig. 3:**
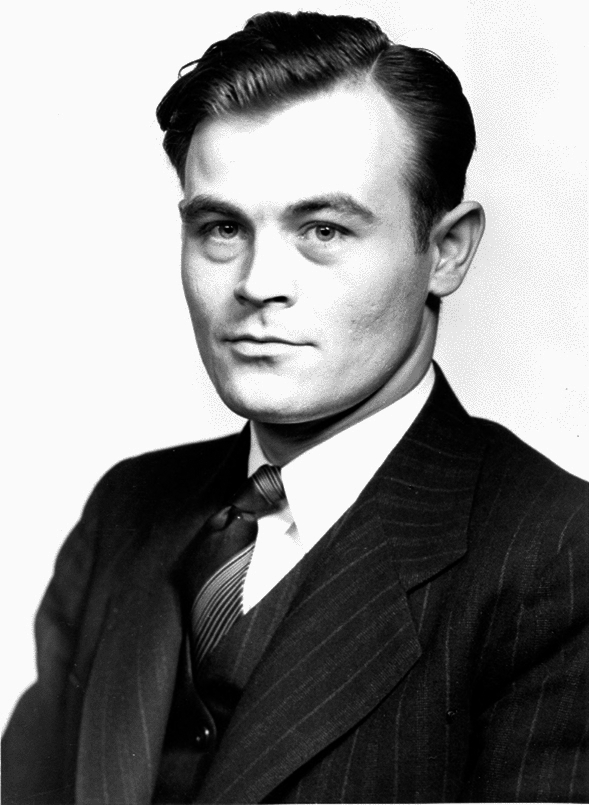
John (“Jack”) Nicholas Wolfe (1910–1974), photograph dated to approximately 1945. Stuckey Herbarium Archives

In her work in Nevada, Beatley was influenced heavily by her doctoral advisor, John (“Jack”) N. Wolfe (Fig. 3), considered by some the Father of Radioecology, the science studying the movement of radioactive substances through the ecosystem, including impacts on humans and other organisms (Seymour 1976; Kangas 2023). He had studied at OSU, where Edgar Transeau and Lawrence Tiffany had become aware of his abilities. He completed both his MS and PhD on the lichens of Ohio, and this work was published as a single monograph by the Ohio Biological Survey (1940).

Transeau hired Wolfe as an instructor in 1937 to assist him with teaching ecology. Upon Transeau’s retirement in 1945, Wolfe assumed responsibility for the ecology program for the next ten years. During this time, in addition to his teaching responsibilities, he did research in vegetation history and bioclimatology, publishing on the Neotoma Valley in the Hocking Hills region of Ohio (Wolfe et al. 1949). He was regarded as an expert mentor  during his time at OSU and supervised fourteen master’s and ten doctoral students. In 1955, Wolfe was invited by Paul Pearson, Chief of the Biology Branch of the AEC, to join it for a two- year period at its headquarters in Washington, DC (Seymour 1976).

During this two-year period, Wolfe became familiar with ecology-related activities in federal government agencies, and became concerned that ecological perspectives were lacking in the nuclear programs (Rothschild 2013). At the end of this period, Wolfe was asked to stay on permanently at the AEC, but he elected to return to OSU. Having returned to academia, Wolfe realized that nuclear technology, if improperly used, could destroy the environment, and this led him to return in 1958 to the AEC to assume the permanent post of Chief of the Environmental Sciences Branch of Biology and Medicine. In this role he was able to shepherd the Commission through challenging times with the goal of adopting a broad ecosystem approach to understanding radionuclides in the environment.

On December 8, 1953, the Atoms for Peace program was presented by President Eisenhower to the United Nations as a peacetime application of using atomic power for civilian projects. Wolfe became intimately involved with this program, and as one example he participated in an extensive environmental study between 1959 and 1962 of a site proposed for using an atomic explosion to create an artificial harbor in coastal Alaska.^9^ This study was probably the very first environmental impact assessment (Rothschild 2013), and it, and numerous political considerations, led to eventual cancellation of the project. The report was made public in a massive 1249-page book that appeared in 1966 (Wilimovsky and Wolfe 1966).

Wolfe continued to advocate for an ecosystem approach to radioecology. In 1960, he undertook a study to examine the impact of radionuclides produced at the AEC site in Hanford, Washington, on the Columbia River Estuary and adjacent ocean waters. In 1963, Wolfe collaborated with ecologist Howard Odum on a project to study irradiation and ecology in a tropical rainforest in Puerto Rico. In 1970, he took a year of administrative leave from the AEC and went to the University of Washington in Seattle to lecture and prepare a textbook on radioecology. Poor health intervened and forced him to retire in 1972 before he was able to finish the project; he passed away in 1974 at age 64. Whether his exposure to radioactive materials or substrates during his career negatively impacted his health is not known.

There is little doubt that Wolfe had a profound impact on Beatley. She had high respect for his abilities as an ecologist, and she valued his mentorship at Ohio State. She also shared his concern that there was a lack of ecological information that would permit wise decisions about projects with potentially broad environmental impacts, and this helped to drive her work at the NTS. Her book, *Vascular Plants of the Nevada Test Site and Central-Southern Nevada* was dedicated to him (Beatley 1976a). Most importantly, at the time Wolfe returned to the AEC, Beatley was again in need of employment.

## New Mexico Highlands University and UCLA

A big transition in Beatley’s professional life took place in early 1959, when she took a position for six months between January to June as research associate in the Department of Biology at New Mexico Highlands University in Las Vegas, New Mexico. The group at Highlands University had already established a track record for their biological investigations at the original Trinity atomic test site in New Mexico,^10^ and had begun field work at the NTS in 1957. Exactly how this six-month appointment at Highlands University came about is unclear, but it seems likely that Wolfe may have recommended her as a research assistant. Given her academic background oriented solely to research on eastern deciduous forests, it may seem odd that Beatley would have accepted a position at Highlands University investigating the ecological impacts on plants from thermonuclear explosions in the deserts of the southwest, but the reality was that she needed employment.

When Beatley joined this group, she was immediately oriented toward the NTS (not Trinity), and the main focus of her six- month appointment was to do preliminary field work with an eye to preparing a contract with the AEC for continued employment. At this time, jurisdiction over research at the NTS had changed from Highlands to the Laboratory of Nuclear Medicine and Radiation Biology at UCLA. At first glance, this would appear to be yet another temporary position like those that characterized the early part of her professional career, because she returned to the University of Tennessee, Knoxville, from mid-1959 to the spring of 1960.

But by the summer of 1960, Beatley returned to the NTS, this time employed by UCLA. The arrangement was to spend part of her time at the NTS, living in Mercury, Nevada, and part of her time at UCLA. The years between 1960 to 1973 provided her with relative stability in her professional life; they represented her longest stable employment, but always as a contract worker, without a permanent academic position. Her initial responsibilities were to set up sample plots, inventory species, assess vegetation, monitor the environment (i.e., take weekly or bi-weekly data measurements on temperature, precipitation, etc.), and describe injury to plants and vegetation from the nuclear detonations. She had assistants and collaborators from time to time as acknowledged in her papers, but correspondence reveals that she was largely a one-woman show.

## Correspondence with Peter Raven

Shortly after arriving for work at the NTS, Beatley began an extensive correspondence with the well-known botanist, Peter Raven.^11^ She had been trained as a plant ecologist and was not familiar with plants of the Great Basin and western desert. She depended upon a trained plant taxonomist, and Raven offered to help Beatley identify plants from the NTS, which included grasses, sedges, rushes and members of Compositae and Leguminosae. He enlisted the help of specialists in other plant groups, such as Rupert Barneby (on *Astragalus*), and Mildred Mathias (on the Umbelliferae). Beatley returned the favor by collecting materials that Raven needed for his studies of *Camissonia* and other members of Onagraceae (especially buds and seeds), and he also wanted seeds from various members of Compositae for chromosomal studies.^12^

The correspondence with Raven began in 1959 when Beatley was wrapping up her six-month position as a research associate with New Mexico Highlands University. By this time, Beatley was working in Mercury at the NTS, and Raven was at the Rancho Santa Ana Botanical Garden (now the California Botanic Garden).^13^ A number of letters exchanged between Beatley and Raven indicated his interest to visit the NTS. Raven had previously requested permission to visit the site in 1959, and Beatley offered to help him, but she was unable to follow through during the summer because he was doing fieldwork in Colombia. She directed Raven to contact Wolfe at the AEC to secure the required permits to visit the restricted test site. This required a great deal of work from her obtaining security clearances, badges, lodging, meals and so on. Raven also wanted to visit the site when seasonal conditions allowed for collecting flowers, fruits, or seeds. The spring of 1964 met all criteria, and Raven finally visited the NTS in mid-April of that year.

In late 1969, Beatley’s work at the NTS started winding down. In early 1971, Raven was about to move to the Missouri Botanical Garden at St. Louis, but he was still interested in receiving seeds of *Oenothera* and *Camissonia* for his studies. Beatley’s time at the test site ended officially in March 1973, but she was back during summers in the mid-1970s, finishing up her work and still obtaining plant specimens and seeds for Raven. In a letter from late March of 1975,^14^ she thanked him for his help over the years, dating back to 1959.

The long correspondence between Beatley and Raven suggests that the exchange was mutually beneficial. From Raven’s perspective, he had found a research colleague who was a competent field collector, a good naturalist observer, and who had access to the NTS. She received help from Raven for the identification of plants in many different families and for making contacts with other taxonomists to help with critical or difficult groups. She needed this taxonomic information to describe the vegetation and infer dynamics of the desert ecosystems. Raven  was impressed with Beatley’s rigor in collecting and identifying plants because, by spring of 1962, he encouraged her to undertake a flora of the NTS.^15^ Trained as an ecologist, not a taxonomist, and being tasked to work in an entirely new flora, she appreciated Raven’s support. She, in turn, was able to supply him with data, observations on pollinators, and collections of Onagraceae for his research programs.

## The NTS Herbarium and the University of Cincinnati

Although Beatley was not trained as a taxonomist, she understood the importance of making voucher collections, dried and pressed plant specimens used to document research activities preserved in a herbarium. A herbarium had been started in the NTS at the town of Mercury by Shields and colleagues from Highlands, but the specimens lacked adequate collection data (Beatley ).^16^ Beatley put the NTS herbarium on a better scientific level, accumulating more than 13,500 specimens by the end of her contract work (Holmgren and Keuken 1974). This collection was the reference set of identified material with which she worked when assessing vegetational patterns and carrying out analyses of vegetation within the overall site, as well as within her own designated permanent plots.

Beatley asserted in a letter to Paul Dunaway and colleagues at the AEC that she originally set up the herbarium at the NTS in 1961,^17^ but this appears not to be the case. As Shields et al. (1958, p. 6) noted, collections dating from 1957 from the NTS were augmented by an additional 122 species that were collected between March and October, 1958. These were prepared as herbarium specimens and were mounted in duplicate or triplicate for herbaria at the NTS and New Mexico Highlands University. The list of species appears in Appendix A in Shields et al. (1958) and includes eight trees, forty-eight shrubs, twenty-five grasses and 157 herbaceous dicots. These collections indicate that the NTS Herbarium existed before the arrival of Beatley at New Mexico Highlands University in 1959, but upon coming to the test site, she discovered that the herbarium specimens were “unaccompanied by field or other data”, which led her to institute improvements.

This initiated a pattern of conflicting interpretations of the NTS herbarium and its contents between Beatley and others in the AEC, and its successor, since January 19, 1975, the Energy Research and Development Administration (ERDA). She regarded the herbarium sheets that she collected as items of “data” belonging to her. Such proprietary interests were not common in the plant systematics community, which held to the view that specimens collected under the auspices of an institution became its property and were permanently deposited there. Ownership of the specimens became a recurring issue between Beatley and her superiors during her time at NTS and beyond.

As her work at the NTS began to wind down, Beatley contemplated the final disposition of the herbarium, which had 13,500 accessioned specimens (Holmgren and Keuken 1974). Another 20,000 duplicates had already been distributed among herbaria at the University of Nevada at Reno, the New York Botanical Garden, Utah State University at Logan, the Rancho Santa Ana Botanical Garden and the University of Nevada, Las Vegas. In a letter in 1970 to Charles Osterberg of the Division of Biological and Environmental Research of the AEC, Beatley requested that specimens of the NTS Herbarium at Mercury be transferred to the US National Herbarium at the Smithsonian Institution. Her letter triggered a response from Osterberg,^18^ copied to John Wolfe and others, suggesting that this action might be premature. Osterberg asserted that the plant collections belonged to the AEC (and ERDA) as long as the agency needed them, and they should remain at the test site where they could be readily accessible for future investigators.

As the AEC transitioned into ERDA and research work at the NTS began to change, Beatley’s contract with UCLA ended in 1972. In January 1973 she accepted a non-tenured position as plant ecologist in the Department of Biological Sciences of the University of Cincinnati. Because the job had been advertised at the Assistant Professor level, she was encouraged to obtain one-third of her salary from another AEC contract to allow her to have an Associate Professor position, which she successfully accomplished, but without tenure and on a five-year contractual  basis. She was eventually successful in being promoted to tenured Professor in 1977, continuing to teach plant ecology and analyzing her NTS environmental data. She retained this position until her death.^19^ Regarding the NTS herbarium, after leaving NTS in 1973 and going to Cincinnati, Beatley took thousands of specimens with her, stating in 1976 in a letter to Paul Dunaway of the Nevada Operations Office (NVOO) that she was still working with them, making an inventory.^20^ She further stipulated that all requests to use the NTS Herbarium should be routed through her in Cincinnati and wanted guidelines for herbarium use to be spelled out concisely. Interestingly, even though she had already moved to Cincinnati, Beatley signed correspondence as “Curator, Nevada Test Site Herbarium,” revealing that she was not giving up on her responsibilities.^21^

In August of 1976 in a letter to Thomas O’Farrell and other administrators,^22^ Beatley took her arguments to a broader level and contended that her research collections at NTS were never under the jurisdiction of the NVOO or Civil Effects Test Office. She also asserted that since her move to Cincinnati in 1973, the plant specimens and all other “data” now belonged to the herbarium of the University of Cincinnati (CINC), with only the six cabinets at Mercury and their contents (approximately 5,000 specimens) constituting the NTS Herbarium. Furthermore, the sheets that she had taken to Cincinnati were deposited personally by her in the National Herbarium (US) at the Smithsonian by late March 1982.^23^ Only twenty-thirty sheets now remain at CINC.^24^

## Scientific Contributions

The original focus for Beatley’s contract work at the NTS was on assessing the ecological impacts on the flora and vegetation deriving from the large number of atmospheric atomic tests. This had several dimensions, some of which were new and challenging to Beatley, and some of which fell within her broader ecological training at OSU with Jack Wolfe and the well-known ecologist Edgar Transeau. But she obviously had to learn a new vascular flora so that the vegetation could be analyzed and basic ecological studies conducted. The existing taxonomic resources at this time consisted primarily of the *Flora of Utah and Nevada* by Tidestrom (1925), *Contributions Toward a Flora of Nevada* by various authors (USDA 1940–1965), Holmgren’s manual for northeastern Nevada (Holmgren 1942), and Linsdale et al. (1952) for part of central Nevada, all of which, although incomplete for the NTS, provided a useful starting point for learning the plants of the region.

The main line of research productivity from Beatley was on eleven checklists and floristic treatments of the NTS and surrounding regions, constituting thirty percent of her total career publications, or thirty-seven in total. She reached out to botanists at Brigham Young University, and formed a strong tie with James Reveal; this was an association that lasted for decades, especially regarding help with the large and taxonomically complex genus *Eriogonum* in the Polygonaceae. She pursued identifications carefully, writing to specialists, and often seeking assistance from Raven. Some collaborations resulted in the coauthoring of taxonomic papers.^25^ Most of this work was a lead-up to her book on the flora and vegetation of the NTS, which represented the most detailed study of the vascular flora and vegetation of any part of Nevada (Beatley 1976a). Many of these data had been published in obscure technical reports (ten in total) from her contracts between UCLA and the AEC, as well as ERDA.

Beatley also published on the effects of radiation on plants and vegetation (1965a, 1965b, 1966), following the earlier investigations by Shields and coauthors at Highlands University (Shields and Wells 1962; Shields et al. 1963) . These early studies had shown that total devastation of plant life occurred one-half mile from ground zero, followed by noticeable, but lessened, damage up to two miles away. Beatley’s further studies continued to confirm these general observations, but the long-term effects of radioactive exposure from the tests were unknown.

**Fig. 4:**
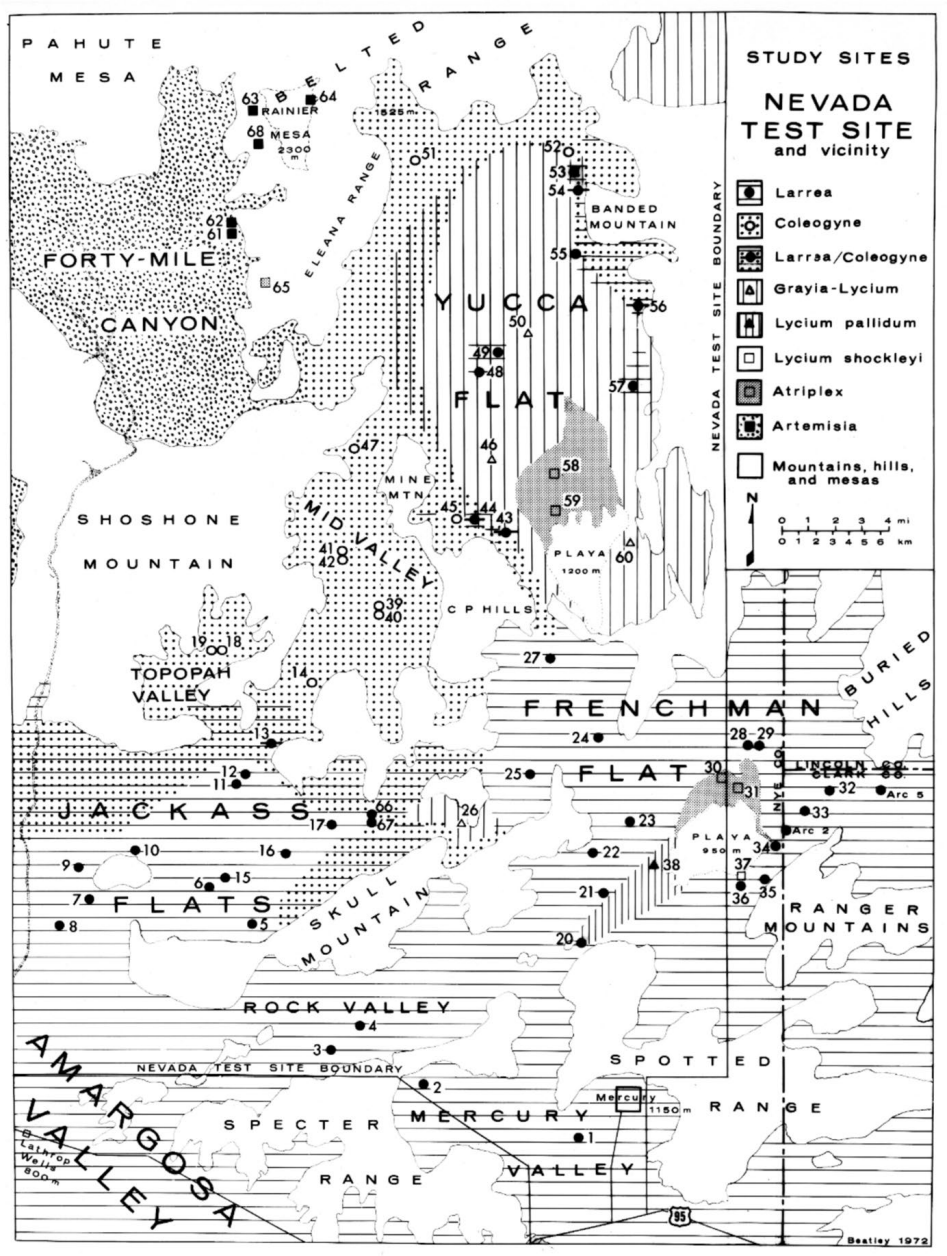
Vegetation of the Nevada Test Site and locations of Beatley’s sixty-eight permanent plots. From Beatley (1974a, p. 246)

Beatley’s main contributions were on the basic ecosystem dynamics within the desert vegetation. With William Rickard she published an estimation of canopy-coverage in the desert shrub vegetation at the NTS (Rickard and Beatley 1965). Beatley’s experience working in Ohio also allowed her to assess the vegetation and produce a map of the entire area (Fig. 4). The NTS consisted of parts of the Great Basin Desert to the north and the Mojave Desert to the south (Shreve 1942), and a transition zone in between, which made it possible to compare patterns among the areas (Beatley 1975, 1976a, 1980).

**Fig. 5:**
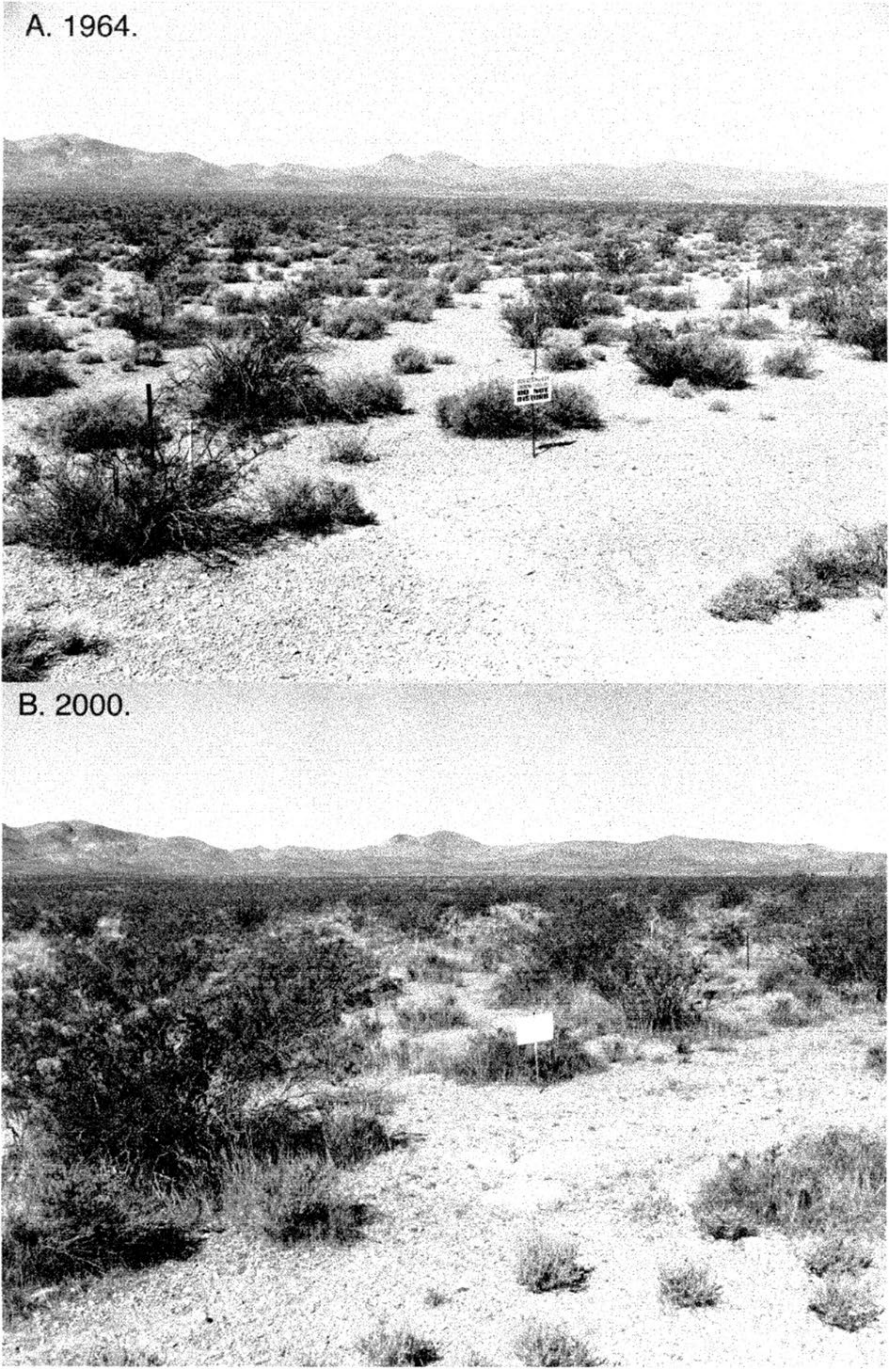
Comparison of *Larrea–Grayia–Lycium* vegetation in one of Janice Beatley’s permanent plots (Number 2) between 1964 (**A**) and 2000 (**B**). From Webb et al. (2001) This is their fig. 4 on unnumbered p. 2

Of special long-term value was Beatley’s establishment of sixty-eight permanent plots, 10 X 10 m, scattered throughout the NTS (Fig. 4). Earlier studies by Shields et al. (1963) had set up fifty plots, but these did not cover the range of vegetation types found in the NTS, nor did they survive. Each of Beatley’s plots was marked with a tall metal stake, which still exists today (Fig. 5). Her methods were to use transect lines to objectively obtain quantitative data for the presence and abundance of all vascular plant species within each plot. In addition, she obtained environmental measurements on precipitation, maximum-minimum air temperatures, soil analyses (moisture, temperature, particle size, pH), gamma radiation, elevation, and rodent captures. All data were put on 250,000 IBM punch cards that were later transferred to magnetic tape and are now on deposit with the US Geological Survey in Reston, Virginia.^26^ The amount of data accumulated was impressive, but no comprehensive synthesis of this information has yet been attempted.^27^

Beatley also published three papers that focused on the impacts of rodents, mostly kangaroo rats (three species of *Dipodomys*), on the vegetation and vice versa (Beatley 1969a, 1969b, 1976b, 1976c). She was aided in the live-trapping by Ernest A. Carl (1964–1965) and John G. Hemington (1966–1968). She demonstrated that the levels of rodent populations fluctuated in response to the abundance of winter annuals that was controlled by precipitation. These studies are of lesser significance than her botanical efforts, but they show her interest in taking as broad an ecological perspective as practicable.

**Fig. 6:**
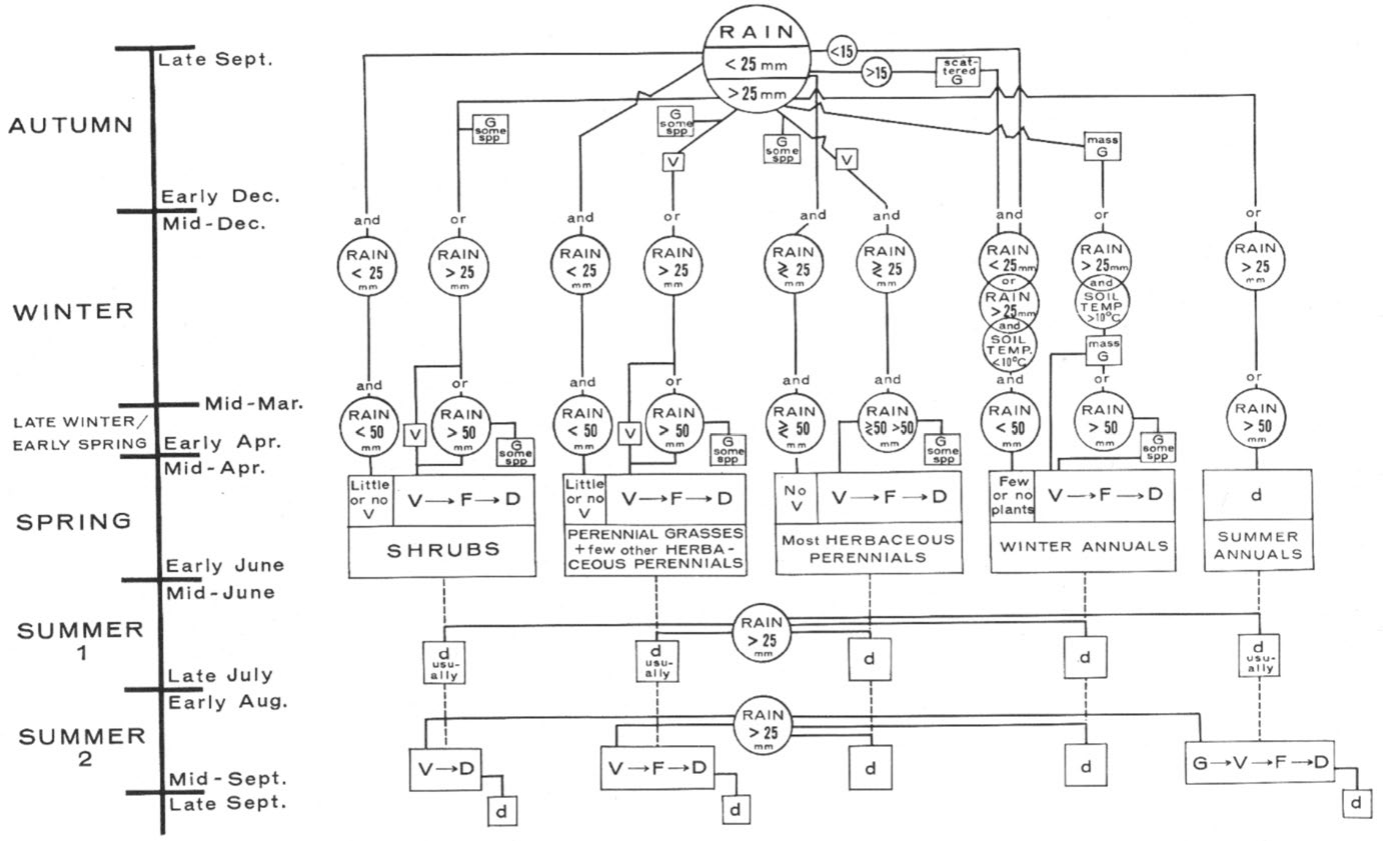
Predictive model as a flow diagram of environmental triggers and phenological events in plants of the Mojave Desert ecosystems in the Nevada Test Site. From Beatley (1974b, p. 859)

One of Beatley’s most significant ecological contributions was to establish a model for the impact of precipitation in the survival of winter annuals (Beatley 1967, 1969a, 1970), and more broadly on all plants of the ecosystem (Beatley 1972, 1974b). She determined that rainfall was the key environmental factor for the appearance of plant diversity, especially heavy rains (>25 mm) between late September and early December. Based on thirteen years of work of sampling in her permanent 68 plots, she offered a predictive model for how different levels of rainfall would impact the survival of different types of plants over a two-year period (Fig. 6).

## Relationships with Administrators

As Beatley continued to work at the NTS, her correspondence reveals a growing frustration with the AEC bureaucracy and professional governmental employees. Part of the problem may have been because Beatley was from academia, and nearly all of the individuals she worked with were governmental bureaucrats as well as men. Many of the letters exchanged between Beatley and her supervisors contained negative references to the abilities possessed by the permanent governmental staff. As she neared completion of her contract work in the early 1970s, she became more confrontational.

Beatley openly challenged the ability of her superiors to make decisions related to the NTS herbarium and the plant collections contained therein. She was highly protective of the herbarium, viewing it as the fruits of her personal labor, and she viewed herself as the only individual within the AEC and ERDA who knew anything about herbaria and how to run one. As such, she wanted to restrict the use of the herbarium to trained personnel and did not hesitate to call out individuals who lacked that training.^28^

Work shifted in the early 1970s at the NTS to determination of rare and endangered plants within the area of the test site, which was federally owned and protected land, as required by the Endangered Species Act of 1973. The work was contracted to a number of private environmental consulting firms for example, Edgerton, Germeshausen & Grier (EG & G), but Beatley disparaged the qualifications of these operatives.^29^ It is not surprising that many of the men with whom Beatley corresponded at this time chose not to deal with her directly, because she had become combative and accusatory; it may be that her personality was beginning to wear on associates within the state and federal governments. In the initial proposals for contract work on locating rare and endangered species within the NTS, Beatley’s work was mentioned, and in fact, she was listed as a participant in the first project, but not conspicuously featured.^30^ She felt overlooked as an essential part of the effort to identify rare and endangered plants at the site.^31^ She found it “insulting” that she was asked to indicate (to Rhoads and others) all rare and endangered species, and her response was: “...my science is not going to be allowed again to be prostituted in the political arena.”^32^

By the late 1970’s, Beatley was taking almost everything as a personal affront.^33^ She became bitter, and there were probably some valid reasons for this. She was a victim on one hand, her expertise being ignored, but the instigator of her own demise on the other. She authored a substantial book on the vegetation of the NTS but was then pushed to the side when the focus shifted to documenting rare and endangered plants. Not only was there a clash between the world of academia and government, but also a conflict between a woman scientist and a cadre of men who were in positions of administrative authority over her; she responded, “frankly, I don't have time or inclination to play games, fight battles, or wage wars for what is patently right.”^34^ This aspect alone might have deterred many scientists from carrying out such research. Potential health hazards from radioactive fallout was yet another problem. The after-effects of these personal conflicts were both profound and long-lasting. In addition, the world had changed by 1974: her mentor and supporter, Jack Wolfe, had passed away, and the AEC had transformed into ERDA and the Nuclear Regulatory Commission.

## Beatley’s Challenge as a Woman in Botany

The challenges that Janice Beatley faced as she sought an academic position can be evaluated in the context of a broader perspective of women in science, and botany in particular. Of all the sciences, botany had always been of interest to women, especially in the 19^th^ century (Rudolph 1982), because they were keen observers of nature (Gianquitto 2007). Women had always been closely associated with teaching their children about the natural world (Kohlstedt 2010), especially plants, and these interests meshed nicely with their role in society. They were encouraged to write about their observations and make drawings and illustrations of the plants. A number of women were married to botanists, and they often assisted in their husband’s research (Stuckey 1992a; Lykknes et al. 2012; Slack 2012). A small subset of these women were able to earn undergraduate degrees in botany transitioning into formal teaching positions at the primary and secondary levels and then at women’s colleges by the late 19th century (Rossiter 1982, pp. 160–217). Less common were opportunities for women to pursue careers in research, especially at major academic institutions (Keller 1983). Government did offer some research opportunities, but these were also few in number.

By the early 1900s, the role of women in the plant sciences began to expand, and they began to make inroads into university research labs in the natural sciences. In genetics, women became involved with research on fruit flies, *Drosophila* spp. (Dietrich and Tambasco 2007), and in the landmark work of Babcock on the plant genus *Crepis* (Smocovitis 2009). This involvement began initially with women serving in clerical roles, typists or as illustrators. In the project on *Crepis*, for example, the women branched out to include seed collecting, plant culturing, and translating scientific texts from the original German and Russian. By the late 1920s, Babcock collaborated with a number of women as students, assistants and colleagues including Priscilla Avery, Lillian Hollingshead, and Margaret Mann. These women carried out significant basic research and data collection (Smocovitis 2009).

Up to the time of the Second World War, the number of women in botany remained fairly constant, but after the start of the war and US involvement, women became widely incorporated into manufacturing, communications, health care, and numerous other positions. Because many men had enlisted to fight in the war, temporary vacancies were available for qualified women, even in academia. In 1942 there were 782 women who were in faculty positions in the biological sciences in US colleges and universities (Rossiter 1995). This number increased to nearly 2,600 by 1946, a year after cessation of hostilities. But as military personnel returned from the war, some 7.8 million of them (mostly men) took advantage of the GI Bill to attend institutions of higher learning (Rossiter 1995, pp. 277–303). Veterans returning from the war typically took four to five years to earn college degrees, resulting in a lag until these GIs entered the workforce.

By 1950, some of these newly-minted college graduates began to trickle into the work place, with some opting to pursue advanced degrees and seek academic positions. More individuals were applying for a relatively static number of academic teaching and research openings. In the 1954–1955 academic year, there were 488 botanical faculty in colleges and universities in the United States, with sixty-eight of them being women or 14% of the total (Rossiter 1995). Competition had dramatically increased at the time Beatley was actively seeking full-time employment; she had graduated with her PhD in 1953, which may have been one reason for her assuming several temporary teaching positions at Ohio State, the University of Tennessee, and North Carolina State College (now North Carolina State University). This carousel of temporary jobs resulted in repeated moves and made establishing any type of research program nearly impossible, and it likely played a role in Beatley accepting a position with the AEC and Highlands University, which took her out to the NTS. In this position, it would be possible for her to set up a longer-term, ecologically oriented research program (it had lasted from 1959 to 1973).

Although Beatley faced many obstacles in pursuing permanent employment, she was inspired and encouraged by the success of two Ohio women academic botanists who served as role models, Clara Weishaupt and E. Lucy Braun. While Beatley was studying at OSU, Clara Weishaupt (1898–1991) was hired as a botany instructor in 1946. Weishaupt was a fixture at Ohio State from 1946–1968 and became curator of the university herbarium in 1949 (Stuckey 1992a, b), publishing several significant taxonomic works (Weishaupt 1960; 1985). Weishaupt and Beatley took several field trips together while the latter was teaching at Ohio State during the 1950s, and they developed a friendship. Both had deep Ohio roots and earned all of their degrees, undergraduate and graduate, at Ohio State. Although neither was trained as a plant taxonomist (Weishaupt had been trained in plant physiology), their employment situations mandated a rapid transition to be able to identify plants correctly, as Weishaupt took over herbarium curatorial duties at Ohio State, and Beatley helped to establish the herbarium at the NTS. It is also likely that the bond between these two women deepened because they were in a field dominated by men. The friendship continued after Beatley left Ohio State, with Weishaupt visiting the NTS in 1964.

The other woman who likely influenced Beatley was E. Lucy Braun (1889–1970) at the University of Cincinnati. Braun had a long and productive career at Cincinnati from 1914 until 1948. She was a plant ecologist in the first wave of women who taught and did research at the university level. Like Weishaupt and Beatley, Braun earned all of her academic degrees at a single institution, in her case, the University of Cincinnati. Braun is regarded as a pioneer in plant ecology, and best known for her 1950 book, *Deciduous Forests of Eastern North America*. She is regarded as one of the most productive women botanists of her time (James et al 1971; Bonta 1991). Beatley was well aware of Braun’s contribution to the field of plant ecology, and as noted by Ronald Stuckey, when Beatley moved to the University of Cincinnati in 1973, she was fulfilling a long-time dream of teaching in the same academic department as Braun (Stuckey 1992a, b). Both women were trained as plant ecologists and shared interests in the eastern deciduous forest, conservation, and habitat preservation (Sicherman and Green 1980; Bonta 1991).

## Conclusion

Janice Beatley was a pioneering plant ecologist who made significant contributions to understanding the ecosystems of the Mojave and Great Basin Deserts of the southwestern United States. Her research provided the most detailed inventory of the flora and vegetation of any part of Nevada, and her environmental data and sampling methods allowed understanding of the precise role of precipitation as a trigger for the development of herbaceous perennials in the region. The permanent plots that she established are still being monitored, providing a view of vegetational change over decades. She accepted the challenge of working in the NTS, a region that was a restricted area, only accessible to individuals with a government permit, which made it an attractive site for ecological investigations. On the other hand, however, the region had suffered massive disturbances from atmospheric and underground nuclear tests. Such a disturbance might have deterred many scientists from carrying out such research, and despite safety assurances by the US government, some risks were still involved. Although Beatley enjoyed some collaborative ventures and received assistance especially from taxonomists like Peter Raven and others, she also met with notable challenges especially in securing permanent employment and her placement in the NTS made her a rarity in the workforce. In the late 1950s, fewer than three percent of the plant scientists working for the Department of Defense were women (Rossiter 1995, pp. 277–303), under whose jurisdiction the AEC fell. Though she made notable scientific contributions, she drew the ire of her co-workers who were mostly men and who dominated the agency. In short, her example shows us once again, how marginalization worked to diminish the work of women in science, and indeed the more independent and forceful in personality, the more this seemed to work against them. Beatley died in 1987 at age 68 of pneumonia.

## Data Availability

No datasets were generated or analysed during the current study.

## Footnotes

1 Their first test was in 1949. See Fehner and Gosling (2000).

2 The incidence of thyroid cancer did in fact increase in neighboring areas for which the US government was finally required to provide financial compensation (www.cancerbenefits.com).

3 Useful summaries of these investigations at the Trinity site were published by Shields and Wells (1962).

4 See for example Shields et al. (1958, 1963).

5 See for example Wallace and Romney (1972).

6 Beatley never married. She had no children.

7 See Beatley (1948). It was published in 1956. And see Beatley (1953), published as Beatley (1959a).

8 Janice C. Beatley (1977). Typescript, University of Cincinnati. [Attached to a letter from J. Snider to Ronald. L. Stuckey, April 18, 1991]. Ronald L. Stuckey Herbarium Archives of The Ohio State University, Columbus, Ohio (hereafter Stuckey Herbarium Archives).

9 See the overview in Wolfe (1964).

10 See for example Shields and Wells (1962).

11 For an autobiography and details of Peter Raven's remarkable career see Raven (2021). The Herbarium Archives at The Ohio State University documents this lengthy record with 117 pieces of correspondence (letters and postcards) between 1959 and 1984 beginning when Raven was finishing his doctorate on *Camissonia* (Onagraceae) at UCLA with Professor Harlan Lewis and continuing through his time at the Rancho Santa Ana Botanical Garden, Stanford University, and the Missouri Botanical Garden (Raven 2021), interrupted only by the years he spent at the British Museum (1960–1961) and in New Zealand (1969–1970).

12 Surprisingly, above-ground nuclear testing was still going on at this time until it was banned in 1962.

13 By the fall of 1962, Raven had moved to Stanford University, and they continued to exchange letters and plant materials, with Raven becoming more interested in pollination biology of *Oenothera*. This lengthy chain of correspondence took place while Raven was at Stanford (1962–1969 and 1970–1971), sandwiched around his sabbatical in New Zealand (1969–1970).

14 Janice Carson Beatley (hereafter JCB) to Peter Raven, March 25, 1975. Stuckey Herbarium Archives.

15 Peter Raven to JCB, March 11, 1962. Stuckey Herbarium Archives.

16 Mercury was the administrative center for the entire site; see Merlin (2016).

17 JCB to Paul Dunaway et al., July 16, 1976. Stuckey Herbarium Archives.

18 Charles Osterberg to JCB, December 2, 1970. Stuckey Herbarium Archives.

19 Janice C. Beatley (1977). Typescript, University of Cincinnati. [Attached to a letter from J. Snider to Ronald. L. Stuckey, April 18, 1991]. Ronald L. Stuckey Herbarium Archives. No evidence exists that her exposure to radioactive fallout at the NTS negatively impacted her health or shortened her life (Simon et. al. 2006), but it is known that women can be more negatively affected by radioactive exposure than men (Olson 2019).

20 JCB to Paul Dunaway et al., July 16, 1976. Stuckey Herbarium Archives.

21 JCB to Paul Dunaway, April 9, 1976. Stuckey Herbarium Archives.

22 JCB to Thomas O’Farrell et al., August 25, 1976. Stuckey Herbarium Archives.

23 JCB to Charles Sheviak, April 13, 1982. Stuckey Herbarium Archives.

24 Eric Tepe, pers. comm.

25 See for example Reveal and Beatley (1971; 1974); Reveal et al. (1973).

26 JCB letter to William Rhoads, June 7, 1976. Stuckey Herbarium Archives.

27 Webb et al. (2003) revisited her plots and sixty-four of them are still intact, making it possible to see changes in the vegetation over more than thirty years (Fig. 5). The plots in the NTS are restricted and accessible, accessible only by special permit, which eliminated destruction of plot labels through vandalism or accidents from visitors.

28 JCB to William Rhoads, September 25, 1976. Stuckey Herbarium Archives.

29 JCB to William Rhoads and Mike Williams, September 13, 1976. Stuckey Herbarium Archives.

30 Attached project proposal to William Rhoads to JCB, April 6, 1976. Stuckey Herbarium Archives.

31 JCB to Douglas James, April 27, 1976. Stuckey Herbarium Archives.

32 JCB to Douglas James, April 27, 1976. Stuckey Herbarium Archives.

33 JCB to Douglas James, May 12, 1976. Stuckey Herbarium Archives.

34 JCB to Douglas James, April 27, 1976. Stuckey Herbarium Archives.
